# An Up-to-Date Overview of Liquid Crystals and Liquid Crystal Polymers for Different Applications: A Review

**DOI:** 10.3390/polym16162293

**Published:** 2024-08-14

**Authors:** Jordi Guardià, José Antonio Reina, Marta Giamberini, Xavier Montané

**Affiliations:** 1 Department of Analytical Chemistry and Organic Chemistry, Universitat Rovira i Virgili, C/Marcel·lí Domingo 1, 43007 Tarragona, Spain; jordi.guardia@urv.cat (J.G.); joseantonio.reina@urv.cat (J.A.R.); 2 Department of Chemical Engineering, Universitat Rovira i Virgili, Av. Països Catalans 26, 43007 Tarragona, Spain; marta.giamberini@urv.cat

**Keywords:** liquid crystals, liquid crystal polymers, supramolecular structures, side-chain liquid crystal polymers, mesogen, columnar mesophase, membranes

## Abstract

Liquid crystals have been extensively used in various applications, such as optoelectronic devices, biomedical applications, sensors and biosensors, and packaging, among others. Liquid crystal polymers are one type of liquid crystal material, combining their intrinsic properties with polymeric flexibility for advanced applications in displays and smart materials. For instance, liquid crystal polymers can serve as drug nanocarriers, forming cubic or hexagonal mesophases, which can be tailored for controlled drug release. Further applications of liquid crystals and liquid crystal polymers include the preparation of membranes for separation processes, such as wastewater treatment. Furthermore, these materials can be used as ion-conducting membranes for fuel cells or lithium batteries due to their broad types of mesophases. This review aims to provide an overall explanation and classification of liquid crystals and liquid crystal polymers. Furthermore, the great potential of these materials relies on their broad range of applications, which are determined by their unique properties. Moreover, this study provides the latest advances in liquid crystal polymer-based membranes and their applications, focusing especially on fuel cells. Moreover, future directions in the applications of various liquid crystals are highlighted.

## 1. Introduction

These days, we as a society are facing one of the most complex and challenging problems of our time: climate change. After the Industrial Revolution at the end of the XVIII century and XIX century, the world consumption of fossil fuels increased dramatically, especially during the last century and at the beginning of the XXI century [1]. As a consequence of this revolution, we have experienced incredible demographic growth. Moreover, the evolution of society has aroused an increase in demands, such as lighting, cooking, space comfort, mobility, and communication, in order to satisfy its necessities. These necessities have also produced an increase in the exploitation of resources worldwide, which has increased the effect of climate change [2].

The excess of fossil fuel consumption has promoted the formation of a large amount of greenhouse gasses (GHGs), which are responsible for global warming [3] and air pollution [4]. Despite the evidence of climate change, there are still people around the globe who are skeptical about it [5]. For this reason, some changes are needed in order to raise awareness in society [2,6,7]. Additional important effects of climate change are the extreme weather events that we are living in nowadays, such as extreme cooling or heating. As a result, an increase in mortality and migration has been noticed in several areas of the globe [8], while this extreme weather has a huge impact on agriculture [9,10,11].

With the aim of facing GHG emissions and climate change, the adoption of renewable energies to phase down fossil fuel energy consumption has aroused an important field to invest in, either to expand the already existing technologies or to research new alternatives and clean energy [12]. From this point of view, fuel cell technology has emerged as an alternative to fossil fuels, as it provides efficient and clean energy production. One can distinguish several types of fuel cells according to the used electrolyte (e.g., polymer electrolyte membrane fuel cells (PEMFCs), molten carbonate fuel cells (MCFCs), solid oxide fuel cells (SOFCs), alkaline fuel cells (AFCs), among many others) [13]. Due to their simple construction, fuel cells can be used for different applications, either as stationary or portable devices. Polymer electrolyte membrane fuel cells are one of the most promising types of fuel cells. Perfluorosulfonic acid ionomer-based membranes are the most commonly used membranes for these types of fuel cells. Nafion^®^ (a registered trademark of DuPont Co., Wilmington, DE, USA) is the most used material for this application, as it presents several advantages, such as having excellent chemical and mechanical stability and high proton conductivity, among others. Nonetheless, it also presents some disadvantages, such as having a high production cost, poor chemical stability at high temperatures, methanol crossover (when operating on a direct methanol fuel cell (DMFC)), membrane swelling, and a huge dependence on proton transport on the presence of water. Water is required to form the conductive pathways for transporting the proton across the membrane and also for transporting the proton itself, as the transport mechanism is explained by the “hopping” mechanism (also known as the Grotthuss mechanism) and electro-osmotic drag [14,15].

For this reason, alternatives to Nafion^®^ have been proposed in the last decades. Some of these alternatives include the modification of the perfluorosulfonic acid chain, which includes partially fluorinated and non-fluorinated acid ionomer-based membranes and the use of polybenzimidazole membranes (doped with phosphoric acid) or alkaline ionomer-based membranes [16]. Liquid crystal polymers (LCPs) have also emerged as alternatives to Nafion^®^ as membranes in fuel cells due to their tuneability and broad range of applications [17,18,19,20]. A liquid crystal (LC) is a molecule that, due to its chemical structure, is able to form a state of matter, the liquid crystal state (also called the mesophase), an intermediate between a liquid and a crystalline solid. These kinds of molecules are called mesogens (or mesogenic molecules). LCPs can be classified into two main groups: main-chain liquid crystal polymers (MCLCPs) and side-chain liquid crystal polymers (SCLCPs), depending on where the mesogen is located (at the main chain or at the side chains, respectively). Liquid crystal polymers, due to their structure, are able to adopt complex organizations by self-assembling.

In the last decades, our group has focused on the development of liquid crystal polymers with the aim of synthesizing tapered side-chain liquid crystal polymers (SCLCPs) for use as membranes for fuel cell applications. For the synthesis of SCLCPs, our group has focused on the use of the tapered (or dendron) unit 3,4,5-tris[4-(n-dodecan-1-yloxy)benzyloxy]benzoate (TAPER) as a mesogen, which has been attached to polyethers, polyamines, or polyoxazolines as a side chain [21,22,23,24,25,26,27,28,29,30,31,32,33,34,35,36]. The presence of this dendron induces the self-assembly of the polymer structure to adopt an inner helical organization of the main chain, thus forming columnar structures, as was established by Percec and co-workers [37,38,39]. Thanks to this self-assembly, an inner channel is formed, which endows the ion transport as a result of the presence of basic heteroatoms (oxygen or nitrogen in the main chain). The presence of the long aliphatic chains at the periphery of the structure increases the hydrophobicity of the material and, at the same time, protects the inner channel against the environment.

The success of LCs and LCPs in emerging technologies could help us shift into a more carbon-neutral society. The production of these alternatives could improve the performance of already studied technologies. Moreover, this would make it possible to move from a fossil fuel-based automotive industry to a fuel-cell automotive industry or even produce more efficient batteries.

The scope of this review is to provide an explanation, classification, and up-to-date overview of LCs and LCPs, with a focus on membranes for fuel cell technology as alternatives to perfluorosulfonic acid-based ionomers. This review is divided into different sections, where the distinct types of LCs and LCPs are described, presenting several examples with a broad range of applications. In the following sections, both established and emerging applications of these advanced materials are presented, including display technologies, optical devices, sensors and biosensors, drug nanocarriers, fibers and fabrics, implants, and prosthetics, among others. Additionally, recent advances in the field of membrane technology for various applications are also presented.

## 2. Liquid Crystals: An Overview

Liquid crystals (LCs) were first discovered by the Austrian botanist Friedriche Reinitzer in 1888 while determining the melting point of cholesteryl benzoate. Since then, liquid crystals have been extensively studied, leading to the development of a wide variety of molecules for different applications such as electro-optical displays [40,41], temperature sensors [42,43,44,45], solar cells [46], and biomedical applications [47,48,49,50], among many others. As everyone knows, there are three main states of matter: crystalline solid, liquid, and gas. These states can be distinguished by the organization and mobility of their molecules. In a crystalline solid, molecules are well-ordered, and the substance has a definite shape and limited volume. Liquids, on the other hand, have a more flexible shape due to the higher mobility of their molecules. Finally, gases exhibit chaotic molecular mobility and have neither a limited volume nor a definite shape. For crystalline solids, X-ray diffraction analysis reveals that their constituents (molecules, atoms, or ions) exhibit long-range periodic order, as they are arranged in a definite pattern, forming a 3D network crystal. As a result, crystalline solids exhibit anisotropy in their physical properties (meaning these properties vary in different directions), whereas liquids and gases are isotropic. Liquid crystals share the properties of both crystalline solids and liquids. In other words, LCs exhibit some positional and orientational ordering while their molecules diffuse through the sample. Therefore, they exhibit anisotropic properties like a crystalline solid but with the fluidity of a liquid.

**Figure 1:**
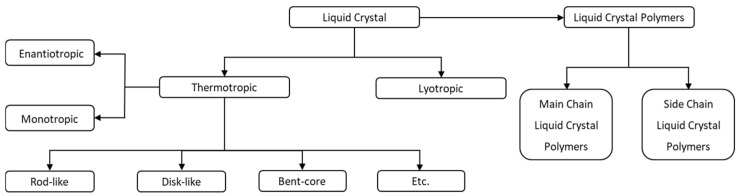
General classification of liquid crystals and liquid crystal polymers.

LCs can be classified based on the formation of the mesophase, which depends on the concentration in a given solution (lyotropic) or by varying the temperature (thermotropic). Among thermotropic liquid crystals, there is a further distinction based on whether the liquid crystal behavior is observed in one direction, either cooling or heating (known as monotropic), or in both heating and cooling (known as enantiotropic). In addition, they can also be classified based on the shape of the molecule (rod-like, disc-like, bent-core, etc.) and the organization in the liquid crystalline phase (smectic, columnar, nematic, cholesteric, blue phases, etc.). Furthermore, polymers can also form liquid crystalline phases when a mesogenic moiety is attached to the main chain (main-chain liquid crystal polymers, MCLCPs) or the side chain (side-chain liquid crystal polymers, SCLCPs). The classification of LCs and LCPs is represented in Figure 1.

### 2.1. Lyotropic Liquid Crystals

Lyotropic liquid crystals are formed mainly by amphiphilic molecules that have a non-polar head and a polar tail. These molecules exhibit a liquid crystal behavior in certain solvents at specific temperatures and concentrations. Lyotropic LC phases can be classified into three main groups: lamellar, columnar, or cubic phases, among others, depending on the positional order [51]. Lyotropic LCs can be found in surfactants like soap and, in nature, can be found in the lipidic bilayer of cell membranes [51,52] or other biomolecules such as DNA, where small fragments can be dispersed in water to present lyotropic LC phases [53]. This class of LCs is used in biomedical applications, such as drug delivery systems [47,48,54] and vaccine platforms [48].

In particular, non-lamellar lyotropic liquid crystals, specifically bicontinuous cubic and inverse hexagonal columnar mesophases, are the most common systems for biomedical applications, as they can be applied either as bulk phases or as colloidal nanocarriers (cubosomes or hexosomes). The bicontinuous cubic phase is a very interesting complex structure consisting of a 3D network with continuous, but not intersecting, hydrophilic sections/water channels. The inverse hexagonal columnar phase is a 2D phase with a hydrophilic inner channel surrounded by a lipidic bilayer. These columns are ultimately organized in a long-range, two-dimensional hexagonal fashion.

Lyotropic liquid crystals have been proven to enhance solubility and slow drug release [55]. Wu and co-workers prepared a lyotropic liquid crystal gel called rotigonite-gel (RTG-gel), obtaining an inverse hexagonal phase, for the treatment of Parkinson’s disease. This gel provided long-acting and slow-release benefits of rotigonite, showing robust, sustained release characteristics as evidenced by the in vitro and in vivo experiments, along with good biocompatibility [56].

One of the challenges of drug administration is the low solubility of these compounds, which directly affects the administration and release of the drug. As we have seen, encapsulating these drugs in lyotropic liquid crystals can improve this issue for oral, injection, and/or transdermal drug delivery. In the field of transdermal drug delivery, studying drug administration across human skin is of great importance. Human or animal skin is often used to study drug diffusion, but this approach involves supply and ethical issues. Lamellar lyotropic LCs, due to their structure, present similarities with biological skin membranes, as they can mimic the stratum corneum lipidic bilayers. Cen and co-workers developed a lamellar lyotropic liquid crystal as an artificial skin membrane and studied the diffusion of four drugs with different polarities. Three artificial skin membranes were synthetized: one containing phytosterols and hydrogenated lecithin, another containing sorbitan stearate and sucrose cocoate, and the third containing cetearyl glucoside. Permeation tests using these synthetic artificial skins mimicked in vitro penetration tests through human skin [57].

Additionally, lyotropic liquid crystals can also be used as sensors due to their stimuli response ability. For instance, Uzun Azar and co-workers developed a cholesteric lyotropic LC-based sensor for detecting toxic gases, such as toluene, phenol, and 1,2-dichloropropane, achieving good results [58]. On the other hand, due to their fluidity, lyotropic LCs can also be used as lubricants. In this regard, graphene oxide (GO) has been studied as a potential material for this purpose since the aqueous dispersion of GO can form a lyotropic liquid crystal phase at concentrations above 5.00 mg/mL. This lubricant is stable for several months and can reduce friction by 37.3%, as reported by Guo and co-workers [59]. Moreover, graphene oxide has also been investigated as a scaffold for the construction of artificial muscles by Gao and co-workers [60]. In their work, they exploited the use of GO sheets in their lyotropic liquid crystal phase to prepare GO fibers combined with the conductive polymer poly(3,4-ethylenedioxythiophene)-poly(styrenesulfonate) (PEDOT:PSS), followed by the reduction of GO to reduced graphene oxide (rGO). The addition of PEDOT:PSS increased the elasticity of the rGO fibers, resulting in outstanding mechanical properties such as Young’s modulus, toughness, elongation-at-break, and exceptional electrical actuation performance. Finally, they demonstrated the potential scalability of the fiber-based artificial muscles and their integration with robotic systems.

### 2.2. Thermotropic Liquid Crystals

**Figure 2:**
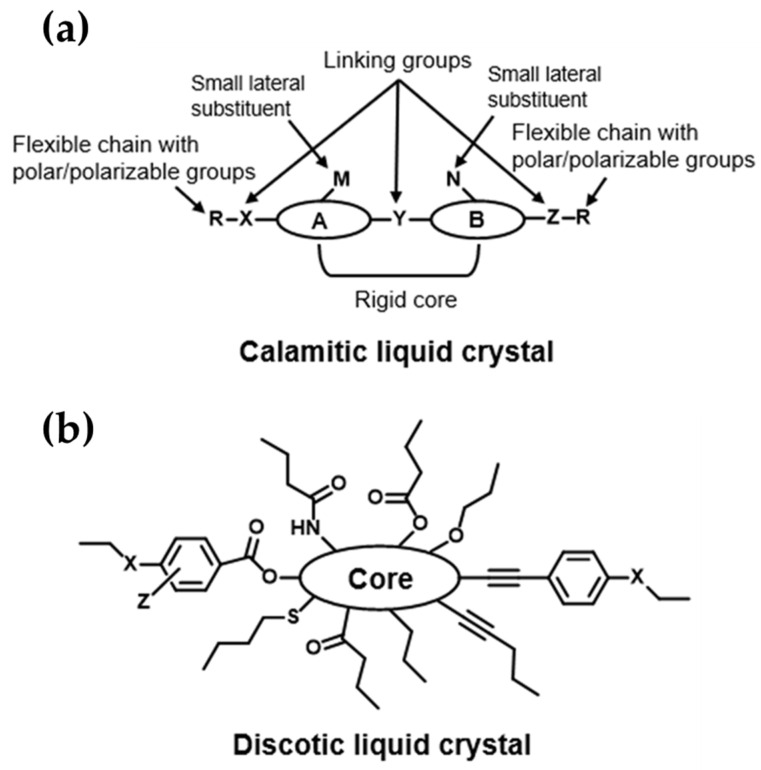
General structures of the two main groups of thermotropic liquid crystals based on their shape: (**a**) calamitic liquid crystal and (**b**) discotic liquid crystal.

When a material exhibits a liquid crystal phase upon varying the temperature, it is referred to as a thermotropic liquid crystal. These mesophases can be obtained by heating a crystalline solid or cooling an isotropic liquid. The transition from a crystalline solid to a liquid crystal phase is called the melting point, while the transition from a liquid crystal phase to an isotropic liquid is known as the clearing point (with the temperature of this transition referred to as the clearing temperature). If a liquid crystal mesophase is obtained by heating and cooling, it is called enantiotropic. Conversely, if the liquid crystal phase is obtained solely by cooling an isotropic liquid or heating a crystalline solid, it is termed monotropic. Not all molecules can form thermotropic liquid crystals; they must meet specific structural requirements, such as having an (often) aromatic rigid core and a flexible peripheral moiety (generally long aliphatic chains). As previously mentioned, thermotropic liquid crystals can be classified into two main types based on their shape: calamitic (rod-like) and discotic (disc-like) liquid crystals. The general structures of these two types of liquid crystals, which will be discussed in the following subsections, are shown in Figure 2.

#### 2.2.1. Calamitic Liquid Crystals

This group of liquid crystals is the most common within the group of thermotropic LCs. They consist of two or more rigid rings in their core (A and B, Figure 2), connected by a linking small group (Y, Figure 2). At each end, there are flexible moieties (R, Figure 2), often composed of linear and/or branched aliphatic chains with polar or polarizable groups. Additionally, calamitic molecules can have small lateral substituents such as -Cl, -Br, -NO_2_, -CH_3_, -OCH_3_, etc., to reduce the symmetry in a controlled manner. Due to the wide variety of different cores, aliphatic ends, small substituents at the core, polar/polarizable groups, and linking groups, calamitic LCs can form different mesophases, including nematic phases and smectic phases. Nematic mesophases (N) are the least ordered mesophases (closest to the isotropic liquid) and are characterized by orientational order without positional order. Nematic mesophases are extensively used in liquid crystal displays (LCD) [61,62]. Smectic mesophases (Sm) present an orientational order, similar to nematic mesophases, but with long-range positional order in one direction. Molecules in the smectic phase are arranged in layers with weak inter-layer interactions that allow them to flow past each other. The presence of more than one mesophase (polymorphism) for a single compound is also possible. For example, da Silva and co-workers synthetized symmetrical 2-methyl[1,2,3]benzotriazole (BZT) derivatives with elongated peripheral units linked via acetylenic triple bonds. Some of these calamitic thermotropic liquid crystals show both nematic and smectic phases upon heating or cooling at different temperatures. Moreover, the bandgaps determined electrochemically for HOMO and LUMO were moderate, indicating the potential use of these compounds for electro-optical applications [63].

Among the nematic mesophases, there is a subclass called the chiral nematic phase or cholesteric mesophase (N*). This LC mesophase is typically exhibited by chiral molecules. It presents a helical structure, which can be visualized as a stack of very thin 2D nematic-like layers, with the orientation of the molecules in each layer twisted slightly with respect to the layers above and below. It is also possible to induce a cholesteric mesophase in non-chiral molecules by adding chiral dopants. These chiral dopants, which may not be LCs themselves, can induce a helical arrangement in thermotropic liquid crystals whose mesophase is otherwise nematic. Smectic mesophases can also exist as a chiral phase, known as the chiral smectic C phase (SmC*), and can be induced by either a single type of molecule or a mixture of compounds [64,65]. Pozhidaev and co-workers reported the synthesis of SmC* ferroelectric liquid crystals (FLCs) by mixing a nematic liquid crystal with chiral non-mesogenic dopants based on terphenyldicarboxylic acid [66]. Nematic LCs were obtained from a eutectic mixture of biphenyl and phenylpyrimidine derivatives. These FLCs were reported to exhibit a broad temperature range (10 to 63 °C), indicating their potential for the design of next-generation photonic devices [67].

In the field of ferroelectric liquid crystals, it is also possible to induce the SmC* phase by adding inorganic dopants. In this context, Pote and co-workers have reported the incorporation of spinel ferrite CoFe_2_O_4_/ZnO (ZCOF) core/shell nanocrystals (NCs) into FLCs. Photoluminescent analysis revealed improved optical emission properties as a direct result of adding ZCOF [68]. Nevertheless, the incorporation of dopants is not the only way to obtain ferroelectric liquid crystals with SmC* phases. For instance, Węgłowska and co-workers have synthetized and characterized nine different compounds of a chiral homologous series of 4′-[ω-(2,2,3,3,4,4,4-heptafluorobutoxy) alkoxy]biphenyl-4-yl-4-(octan-2-yloxy)benzoates, which contain fluorine or chlorine atoms in the lateral position of the rigid core with a chiral terminal octyloxy-chain, and exhibit chiral SmC* phases. According to their study, transductors based on these materials could form new classes of optical sensors suitable for numerous applications [69].

Moreover, thermotropic LCs with cholesteric mesophases have potential uses as optical sensors for biomedical applications, as they are known to enable the optical amplification of biological interactions [70]. For instance, Wu and co-workers reported the preparation of an LC-based single-substrate biosensor by spin-coating a thin film of the same LC onto a dimethyloctadecyl[3-(trimethoxysilyl)propyl]ammonium chloride (DMOAP)-decorated glass slide [71]. This spin-coating method allowed them to control the film thickness with precision. Using this biosensor, they reported an improvement up to two orders of magnitude in the limit of detection (LOD) for bovine serum albumin (BSA), reducing the LOD from 10^−5^ g/mL to 10^−7^ g/mL for a 4.2 µm thick film; and even to 10^−8^ g/mL when the film thickness was reduced to 3.4 µm.

#### 2.2.2. Discotic Liquid Crystals

**Figure 3:**
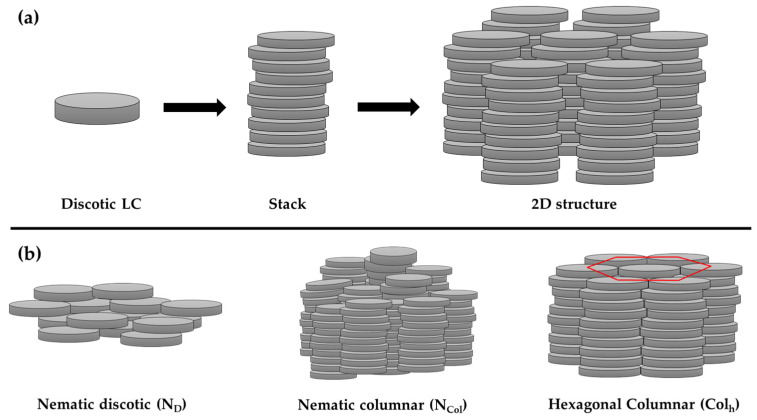
(**a**) Example of self-assembly and self-organization of discotic liquid crystal molecules in columnar mesophases. (**b**) Example of different mesophases presented by discotic molecules. The red circle in the hexagonal columnar (Col_h_) mesophase shows the organization of the columns in hexagonal structures.

Discotic liquid crystals are formed, as the name suggests, by disc-shaped molecules. These types of LCs often spontaneously self-assemble into stacks due to π-π interactions, which eventually self-organize, forming 2D structures (Figure 3). Discotic LCs can form various mesophases: nematic, smectic, columnar, and cubic. Smectic and cubic phases are very rare, while columnar mesophases, which can be easily oriented homeotropically, are the predominant structures, followed by nematic mesophases. However, disc-like molecules are not the only ones that present columnar structures; as mentioned in the previous section, lyotropic liquid crystals can also form columnar structures. Moreover, dendrimers, star-shaped molecules, and even rod-like molecules can exhibit thermotropic columnar phases. For instance, Carrasco and co-workers prepared red near-infrared (NIR)-emissive metal cluster compounds with the general formula Na_2_Mo_6_X_8_^i^ Cl_6_ (X^i^ = Cl or Br), complexed with LCs containing crown ethers [72]. The complexation was made possible by the well-known interaction between sodium cations (Na^+^) and the oxygen atoms of the crown ethers. The prepared hybrid materials exhibited hexagonal columnar mesophases over a broad temperature range. In another example, Labov and co-workers synthetized star-shaped LC molecules as nano-reservoirs for small acceptors based on a 1,3,5-substituted benzene core with either oligo(phenylene vinylene) or oligothiophene arms [73]. The small acceptor was 2,4,7-trinitrofluorenone. X-ray scattering and solid-state NMR analysis confirmed the host–guest complexation for up to three guest molecules per LC-host. In addition, differential scanning calorimetry (DSC) analysis and X-ray scattering confirmed that these materials exhibit hexagonal columnar mesophases at high temperatures.

It is very common to confuse the terms discotic phase and disc-shaped molecules. Therefore, it is important to remember that it is the molecule that is discotic, not the mesophase, which can be nematic, columnar, lamellar, etc. Among the mesophases exhibited by discotic mesogens, the discotic nematic mesophase (N_D_) is the least ordered mesophase and least viscous, similar to calamitic molecules. It allows for translational and rotational freedom around its short axis, but on average, the disk-like molecules are oriented in a preferred direction (Figure 3). The nematic columnar (N_col_) mesophase, on the other hand, is characterized by a columnar stacking of the discotic mesogens, but these columns do not form 2D structures. As a result, the columns lack positional order but exhibit orientational order. Similar to calamitic liquid crystals with nematic mesophases, LC materials that exhibit discotic nematic phases can be used for display applications.

In this context, Dhingra and co-workers developed a disc-like molecule based on a tri-alkynyl benzene core, which exhibited a nematic discotic mesophase at room temperature. The results of their work suggest that this material could be advantageous as a pure deep-blue emissive component for organic light-emitting diodes (OLEDs) [74]. Moreover, disc-like molecules can also be used as solid-state solar thermal fuels (SSTFs), as demonstrated by Gupta [75]. Materials with discotic nematic mesophases were obtained by incorporating a tetra-ortho-fluoro/chloro azobenzene arm into a triphenylene-based LC moiety. The resulting compounds showed excellent photoswitching, photostability, and photocyclability for SSTF applications. The advantage of this system lies in the formation of the discotic nematic mesophase, even at sub-zero temperatures, making it more efficient than traditional solar panels, which are less effective at such low temperatures.

As mentioned above, discotic LCs can organize into columnar mesophases, which eventually self-organize into various 2D lattices. These 2D columnar mesophases can be classified into several types, with the most important being the columnar hexagonal mesophase (Col_h_), the columnar rectangular mesophase (Col_r_), and the columnar lamellar mesophase (Col_L_). Columnar hexagonal mesophases are characterized by a hexagonal arrangement of the columns. The planar space group for this type of mesophase is P6/mm. When analyzing a discotic liquid crystal compound by X-ray diffraction (XRD), in the small-angle region, three sharp peaks can be distinctly observed with spacings in the ratio of 1: 1/√3: 1/2, corresponding to the lattice planes indexed as (1 0 0), (1 1 0), and (2 0 0), respectively, along with a broad peak in the wide-angle region [32]. Columnar rectangular mesophases are characterized by a π-π stacking of the aromatic cores of the molecules, tilted with respect to the column axis and packed in a rectangular fashion. However, it is possible to observe a transition from a columnar rectangular mesophase to a columnar hexagonal mesophase as the aliphatic side-chain lengths increase. Finally, columnar lamellar mesophases are characterized by the stacking of discotic mesogens to form columns, but instead of forming an organized 2D structure, the columns are arranged in layers where they can slide across the layer without possessing translational order.

The efficient capture, transfer, and storage of solar energy represent a fascinating and challenging area of research today. In this context, within the field of discotic columnar mesophases, Mu and co-workers synthesized polymeric supramolecular columns bioinspired by the purple photosynthetic bacteria and its natural light-harvesting mechanisms [76]. A discotic molecule based on non-planar tricyanotristyrylbenzene (TCS) was introduced to a polyacrylate backbone as pendant groups. The resulting polymeric liquid crystals exhibited a hexagonal columnar mesophase across a temperature range of 0 to 130 °C. Moreover, the columnar assembly of the material allowed for additional control over the energy transfer process. Additionally, Sharma and co-workers prepared symmetrical calix[4]pyrrole-based liquid crystals functionalized at the para position with urea substituents, which exhibited a hexagonal columnar mesophase at room temperature [77]. Films made from this material showed a suitable optical energy bandgap, along with favorable absorbance and an extinction coefficient. The findings in this work suggest the potential of these films as an eco-friendly optical window layer in thin-film solar cells. Moreover, De and co-workers envisioned the synthesis of a cyanovinylene-integrated pyrene-based discotic liquid crystal for solar cell applications using a minimalistic design strategy. The resulting material exhibited a room-temperature columnar hexagonal mesophase and a narrow bandgap for efficient semiconducting behavior. Furthermore, it showed an elevated charge extraction ability from contact electrodes at a low voltage, achieving an electrical conductivity of 3.22 × 10^−4^ S/m, the highest reported value for any pristine discotic LC film in a vertical charge transport device [78].

In the field of ion-conductive materials, discotic liquid crystals have aroused a great interest in the formation of electrolytes for energy storage applications due to their controllable ion channels. Notable ionic conductivity has been observed in LC materials involving non-covalent two-component self-assembly interactions of salts and polar mesogenic molecules [79]. For instance, Suwa and co-workers designed supramolecular discotic LCs with columnar mesophases driven by dipole–ion interactions [80]. In their study, they prepared self-assembled columnar structures by ion–dipole interactions between benzonitrile derivatives (*N*-(4-cyanophenyl)-3,4,5-tri(dodecyloxy)benzamide and 4-cyanophenyl 3,4,5-tri(dodecyloxy)benzoate) and imidazolium bromide-based ionic liquids. During mesomorphic characterization, the benzamide-based benzonitrile did not exhibit a liquid crystal behavior on its own, but when combined with the imidazolium bromide-based ionic liquids, it formed columnar mesophases, as evidenced by polarized optical microscopy (POM) and X-ray diffraction (XRD). The location of the ionic liquids at the center of the self-organized columns imparts anisotropic 1D ion-conductive properties to these materials. The ionic conductivities evaluated by electrochemical impedance spectroscopy (EIS) revealed greater conductivity for measurements taken parallel to the column axis compared to those taken perpendicular to it, which is attributed to the hindering effect of the insulating alkyl chains.

As noted, discotic liquid crystals can be used in a wide range of applications, from optoelectronic to ion-conductive materials for energy storage, among others. Behera and co-workers developed room-temperature LCs with columnar mesophases based on perylene-bisimides (PBIs) for use as a novel corrosion-resistant surface film for mild steel (MS) [81]. The potential of these discotic liquid crystals is attributed to their hydrophobic nature due to the presence of aliphatic side chains and their ability to form a protective coating on metal surfaces. The obtained materials exhibited a columnar rectangular mesophase up to 360 °C, including at room temperature. The high isotropization temperature can be attributed to the strong core–core interactions between the aromatic cores. The corrosion inhibition of the films produced with this compound was evaluated by potentiodynamic and EIS at room temperature on MS in 1 M HCl. The films were found to be highly effective corrosion inhibitors, achieving a maximum inhibitor efficiency of 76%.

Artificial muscles are an emerging research field within the realm of liquid crystals, as discussed in the previous section on lyotropic liquid crystals. Recently, columnar materials based on discotic molecules have also aroused great interest in the field of artificial muscles. For example, hemiphasmidic side-chain liquid crystal polymers based on a polycyclooctene backbone and dicyanodistyrylstilbenzene (DCS) rod-like side-chain mesogens bonded in series to a bulky fan-like tail group, synthetized by Yang and co-workers, demonstrated excellent properties when evaluated for use as artificial arm [82]. They present a hexagonal columnar mesophase with three distinct stages: first, the “frozen state”, below the glass transition temperature (T_g_ = 45 °C), where columnar assemblies are rigid; second, the “transition zone”, from T_g_ to 80 °C, where chain segments relax; and third, the “soft state” above 80 °C, where chain movements are active, and the columnar assemblies become very soft. This novel hemiphasmidic side-chain liquid crystal polymer exhibits a unique “breathing” motion that results in negative thermal expansion of the columnar lattice, mimicking the elongation and contraction of skeletal muscles. When heated to 150 °C, the material demonstrates an elongation rate of 118%, comparable to that of real muscle fibers (<120%).

## 3. Liquid Crystal Polymers

**Figure 4:**
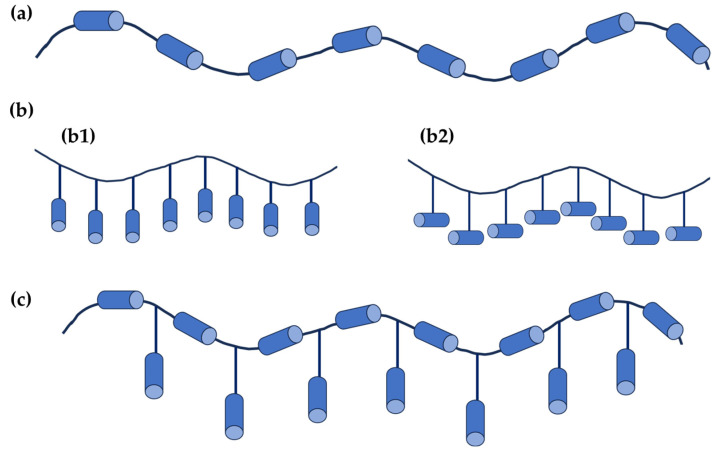
Schematic illustration of (**a**) main-chain liquid crystal polymers (MCLCPs); (**b**): (**b1**) side-chain liquid crystal polymers (SCLCPs) with longitudinally attached rod-like mesogen, (**b2**) SCLCPs with laterally attached rod-like mesogen; and (**c**) main-chain/side-chain liquid crystal polymers (MCSCLCPs). Blue cylinders represent a mesogen.

Liquid crystal polymers (LCPs) attracted great interest following their discovery by Vorländer in 1923 [83]. Their wide range of applications—including separatory membranes, electrolytes, photoelectric conversion, and high-performance engineering plastics, among others—further extends the already broad applications of non-polymeric liquid crystals. LCPs can be prepared using two primary approaches: attaching a mesogenic moiety to a polymer backbone through a post-polymerization reaction or directly polymerizing a mesogenic monomer. Regardless of the preparation method, liquid crystal polymers can be classified based on the position of the mesogen. Therefore, LCPs are categorized as main-chain liquid crystal polymers (MCLCPs) when the mesogen is incorporated into the polymer’s main chain, side-chain liquid crystal polymers (SCLCPs) when the mesogen is linked to the polymer backbone as a side chain, and main-chain/side-chain liquid crystal polymers (MCSCLCPs) when mesogens are present both as the side-chain and within the polymer main chain [84]. A schematic representation of these types of LCPs is depicted in Figure 4.

In a given SCLCP, when the mesogenic unit is rod-like, it can be linked to the main chain in two different ways: longitudinally (end-on, Figure 4(b1)) or laterally (side-on, Figure 4(b2)). It is crucial for SCLCPs to have a spacer between the polymer backbone and the mesogenic moiety. This spacer allows interactions between mesogens, leading to the formation of self-assembling structures and enabling the formation of mesophases. The flexibility introduced by the spacer allows SCLCPs to form different mesophases depending on the type of attached mesogen. For instance, SCLCPs with rod-like mesogens can self-assemble into nematic, smectic A (SmA), smectic C (SmC*), and cholesteric mesophases, whereas SCLCPs with discotic mesogens can form columnar nematic (N_col_), hexagonal columnar (Col_h_), rectangular columnar (Col_r_), and discotic nematic (N_D_) mesophases due to the π-π interactions between the disc-like moieties.

MCSCLCPs are composed of a MCLCP with side-chain mesogens. These side-chain mesogens can be attached directly to the mesogenic units of the backbone or the spacers between the main-chain mesogens.

**Figure 5:**
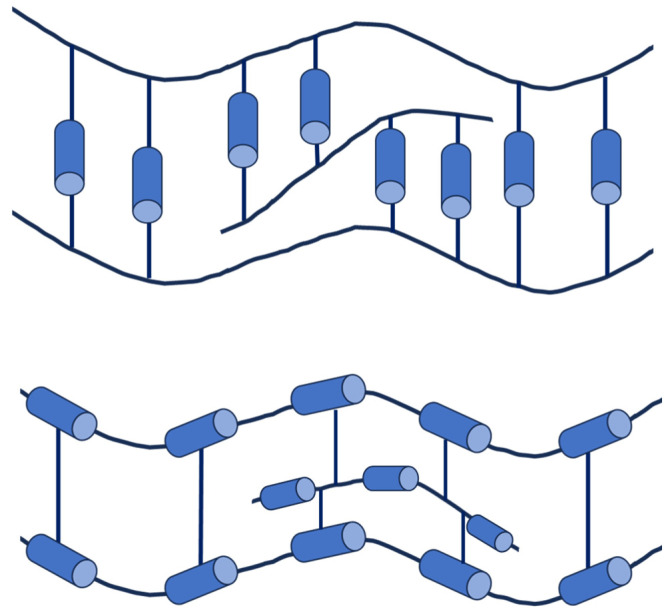
Schematic representation of liquid crystal networks.

In addition to this classification, LCPs can also form LC thermosets or LC elastomers by the cross-linking of polymer backbones with mesogenic molecules (Figure 5). These types of LCPs can be obtained either by the direct polymerization of a mesogenic monomer or by cross-linking MCLCPs.

For example, Vita and co-workers evaluated the liquid crystalline order during the curing of reactive thermotropic liquid crystal macromonomers based on phenylethynyl-terminated biphenol and naphtalenediol model units for the synthesis of LC thermosets. Their findings suggest that the curing mechanism may involve both chain extension and cross-linking, neither of which occurs until 300 °C. Prior to the curing process, both model compounds exhibited nematic mesophases; however, after curing, this mesophase was no longer observed. Instead, an isotropic behavior was observed according to polarized optical microscopy. This behavior differs from that observed with higher molecular weight oligomeric macromonomers, where the nematic mesophase was retained after curing. The authors attribute this difference to the relatively low molecular weight of the model macromonomers [85].

LC elastomers are a class of moving polymers suitable for various applications, such as converting external stimuli to mechanical actions. Pei and co-workers, for example, prepared liquid crystal elastomers with exchangeable covalent bonds, a type of bond used in the preparation of vitrimers. In this work, they used an epoxy-terminated biphenyl mesogen and decanedioic acid as a cross-linker. As a result, these LC elastomers can be reshaped or reprocessed at temperatures above the topology-freezing transition temperature (T_v_) since below this temperature, exchange reactions become extremely slow, and the material behaves like a conventional covalently bonded thermoset [86].

MCLCPs are used in several applications, such as fiber manufacturing, due to their excellent mechanical properties and the anisotropy of their LC phases. In the production of LC fibers, the most widely used MCLCP is a polyaryl amide, commonly known as Kevlar (DuPont), because of its outstanding mechanical properties. However, the LC properties of these materials are only observed in solution, as they have high melting points—above the decomposition temperature—and limited polymer chain mobility. One strategy to reduce the melting temperature and improve both the polymer’s performance and its LC properties involves replacing the polyaryl amide core with an aromatic polyester structure. This structure was further modified by incorporating flexible aliphatic groups into the backbone, adding substituents to the aromatic core, or substituting the central benzene cores with other rigid structures (biphenylene and naphthalene groups). These modifications resulted in a lower melting point, along with the polymers that exhibit low melt viscosity, high heat resistance, high tensile strength, high modulus, and low thermal expansion [84].

More recently, following this approach, Orodepo and co-workers synthesized a series of MCLCPs incorporating a biphenyl mesogen and flexible alkylene spacers in the backbone. Additionally, these polymers had non-mesogenic pendant segments, such as alkyl, polyethylene glycol (PEG), or fluoroalkyl groups, attached to the polymer backbone. These polymers were obtained via melt polycondensation and organized in a zig-zag pattern, resulting in the formation of a smectic mesophase [87]. Wang and co-workers also evaluated the influence of the E/Z configuration on the phase behavior of similar biphenyl-based MCLCPs. In their work, they concluded that the Z configuration did not exhibit a liquid crystalline mesophase, whereas the polymer with an E configuration showed two mesophases (rectangular columnar and smectic) at different temperatures. In this study, the MCLCPs were obtained by melt polycondensation of a biphenyl-based monomer and a tetraphenylethylene-based monomer, obtaining a copolymer [88].

Side-chain liquid crystal polymers can be categorized into two groups based on the shape of the attached mesogenic side chain being either rod-like or disc-like. Similar to low molecular weight calamitic liquid crystal molecules, SCLCPs with rod-like side chains can adopt different mesophases, with the nematic and smectic (A or C) mesophases being the most common [89]. As with the nematic phases in low molecular weight LC molecules, the addition of a chiral mesogen to the polymer main chain can induce chirality in the final LCP, leading to the formation of cholesteric and smectic C mesophases [90].

Recently, in an effort to explore novel smart multifunctional materials, Alaudin and co-workers proposed the synthesis of a new set of block and statistical liquid crystal copolymers based on a light-responsive mesogenic group, polar sulfonic groups, and methyl methacrylate (MMA) groups, synthesized using reversible addition-fragmentation chain-transfer (RAFT) polymerization. Structural and thermal characterization, along with a conductivity study, revealed ionic conductivities in the range of 10^−6^ S/cm, which presents a remarkable conductivity in anhydrous conditions. The light-responsiveness and conductivity observed in these polymers open the possibility of developing polymer electrolytes based on these copolymers for energy storage and energy conversion applications [91].

Regarding disc-like SCLCPs, self-assembling phenomena are observed similar to those in the disc-like low molecular weight molecules discussed earlier. The columnar organization is predominantly observed in these polymers, thanks to the π-π interactions between the mesogenic units. The polymer main chain, the discotic mesogen, the flexible spacer, and the final aliphatic tail play key roles in the self-assembling process. The columnar organization of SCLCPs makes them suitable materials for preparing membranes, where the path is only 1D governed due to their high order and good chemical stability. Thus, for example, Bogdanowicz and co-workers, in work carried out within our research group, reported the synthesis of dendronized liquid crystal polymers based on a poly[2-(aziridin-1-yl)ethanol] (PAZE) modified with the dendron 3,4,5-tris[4-(n-dodecan-1-yloxy)benzyloxy]benzoate [23,29,92]. These SCLCPs were bioinspired by the structure of the tobacco mosaic virus (TMV), whose helical assembly creates an inner channel within the tobacco mosaic virus columnar organization. The helical structure of the PAZE-modified polymers enabled the assembly of membranes for proton transport applications, where protons can be conducted through the membrane’s columnar organization thanks to the presence of basic heteroatoms (nitrogen) in the inner channel.

More recently, Yang and co-workers designed the synthesis of dendritic fluorescent LCPs using polystyrene as the main chain and tetraphenylethylene as the mesogenic unit, linked with different flexible alkyl spacers of 2, 4, 6, 8, or 10 methylene units. The resulting LCPs exhibited nematic columnar mesophases for shorter spacers (2, 4, 6, and 8 methylene units), while the longer spacers (10 methylene units) led to hexagonal columnar mesophases [93]. Bi and co-workers used a similar approach in designing SCLCPs for optoelectronic applications. They used a triphenylene mesogenic unit as a monomer with a fluorine atom at the end of the alkoxy flexible long chain. After polymerization, the obtained LCPs exhibit hexagonal columnar mesophases [94]. These LC polymers and their derivatives hold potential for use in optoelectronic and solar cell applications [95,96]. Thanks to their fluorescent properties, triphenylene derivatives can also be used as ink with “special” capabilities. In this sense, Li and co-workers reported the preparation of a uniform colloidal dispersion based on LCP rods. In their study, they used triphenylene units as side-chain mesogens to prepare different homopolymers and random copolymers with columnar structures, which were synthesized via RAFT polymerization. The addition of two surfactants to the system resulted in colloidal dispersions with a high uniformity, regular shape, and good colloidal stability. The aforementioned “special” capabilities of this ink derive from photo-induced fluorescent enhancement (PIFE) when irradiated with a UV lamp. As a result, paper impregnated with this ink is invisible to the naked eye but can become readable under UV light (365 nm). These findings demonstrated intriguing PIFE properties and an accompanying memory effect, making these dispersions suitable for advanced colloidal inks used in high-level encryption and secure document delivery [97].

## 4. Liquid Crystal Polymer-Based Membranes

Since the beginning of times, biological membranes have been part of all living organisms for millennia, including structures like the lipidic bilayer of cellular membranes, mitochondrial membranes in eukaryotic cells, and liposomes and/or lysosomes, among others [98]. However, the world of membranes extends beyond these natural examples. Synthetic membranes have aroused great interest, especially following their large-scale industrialization in the 1960s. These synthetic membranes are widely used in several applications, such as wastewater treatment [99,100,101,102,103], gas separation [99,100,103,104], CO_2_ capture [105], hydrogen production [106], biomedical applications [107,108,109], and polyelectrolytes [104,105,110,111], among others.

One of the key challenges in membrane technology is achieving uniform pore size. Uniformity is crucial for effective separation, as the lack of uniformity in pore size can restrict molecular selectivity, thereby impacting the membrane’s performance and separation capability.

To address this issue, nanostructured polymer membranes based on self-assembling materials have gained significant interest in recent decades. Liquid crystal membranes derived from thermotropic and lyotropic liquid crystals have become a particularly promising area of research. Nevertheless, the use of these LCPs is not the only method for the formation of liquid crystal-based membranes. Nanostructured membranes can also be fabricated using polymerizable liquid crystals, which are later polymerized and cross-linked after achieving the desired organization or alignment. As a result, membranes with narrow pore-sized distributions, high pore densities, and precise control over functionality and pore size can be prepared [112]. For instance, Suzuki and co-workers developed functional nanoporous membranes based on photocleavable columnar LCPs. In this work, they used a taper-shaped molecule that, in combination with lithium triflate, could adopt a column organization. Furthermore, the photocleavable moiety consisted of an *o*-nitrobenzyl ester that is linked to a dendritic, polymerizable hydrophobic segment containing vinyl groups and a hydrophilic tris(oxyethylene) chain. After the formation and correct alignment of the columns, the material was polymerized via acyclic diene metathesis (ADMET), resulting in a polymer membrane. Finally, the photocleavable linker was removed by UV irradiation, leading to the formation of ordered nanopores [113].

More recently, Lugger and co-workers prepared nanoporous films with photoswitchable absorption kinetics using a similar approach. Thus, a wedge-shaped azo derivative, 4-((2,3,4-tris(undec-10-en-1-yloxy)phenyl)diazenyl) benzoic acid, formed a 3:1 columnar supramolecular complex with a tris-benzimidazolyl benzene core (TB). After polymerization and the removal of TB, nanoporous films were obtained. The trans-to-cis photoisomerization of the azo groups enabled a controlled absorption of rhodamine 6G in the cis-enriched films. This absorption could be modulated by irradiation due to the isomerization of the azo moieties within the nanoporous structure [114].

### 4.1. Lyotropic Liquid Crystal Polymer-Based Membranes

As previously discussed, lyotropic LCs are useful materials for the preparation of membranes used in biomedical applications and drug delivery systems [47,48,54,55,56,115,116]. A significant challenge in membrane technology applications is fouling, primarily caused by proteins and other biomolecules. Lyotropic liquid crystals have proven to be suitable materials for addressing the fouling issue. For instance, Saadat and co-workers developed a thermoresponsive lyotropic liquid crystal block copolymer with potential applications in protein purification and the removal of viruses and bacteria, showing excellent anti-fouling properties [116]. Furthermore, Yue and co-workers explored the encapsulation of a dendritic antimicrobial peptide combined with gold nanoparticles within a lyotropic liquid crystal polymer for treating surgical incision infections. Their study reveals that this drug delivery system eradicated up to 99% of bacteria in wounds while significantly enhancing wound healing [115].

### 4.2. Thermotropic Liquid Crystal Polymer-Based Membranes

Liquid crystal membranes with controlled nanostructures can be formulated using thermotropic LCPs due to their self-assembling ability. Nevertheless, not all thermotropic LCPs are suitable materials for the preparation of LC membranes. Side-chain liquid crystal polymers present unique self-assembling characteristics that make them excellent candidates for membrane assembly. In particular, liquid crystal polymers with smectic mesophases open the possibility of using 2D conducting channels for the transport of different ionic species, as the formation of this mesophase enables the presence of 2D conductive paths. On the other hand, SCLCPs based on discotic mesogens that form columnar mesophases provide 1D conducting paths. Both types of materials are excellent candidates for use in ionic transport applications. For instance, Zhang and co-workers prepared SCLCPs with smectic mesophases, specifically LC ionomers bearing benzoxazole-based mesogenic side chains and their membranes. These polymers exhibited enantiotropic smectic C mesophases [117]. In this work, the heterocyclic mesogenic compound 6-[4-(5-chloro-2-benzoxazolyl)phenoxy]-1-hexanethiol (CBPHT) was synthesized as a side-chain mesogen and grafted onto commercial polyepicholorohydrin (PECH) as the main chain. Membranes prepared with these materials exhibited high thermal stability, acceptable mechanical properties, and reasonably high ion conductivity, indicating their potential application as polymer electrolyte membranes.

**Figure 6:**
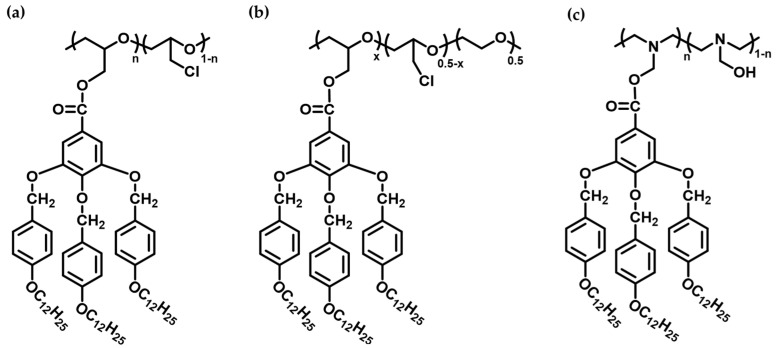
Chemical structure of SCLCPs modified with the dendron 3,4,5-tris[4-(n-dodecan-1-yloxy)benzyloxy]benzoate (TAPER): (**a**) poly(epichlorohydrin) (PECH), (**b**) poly(epichlorohydrin-co-ethylene oxide (PECH-co-EO), and (**c**) poly[2-(aziridin-1-yl)ethanol] (PAZE).

In work conducted by our research group, commercial PECH was used to build up membranes with columnar structures intended for proton transport applications, such as in PEMFC. Thus, PECH was modified with the dendron 3,4,5-tris[4-(n-dodecan-1-yloxy)benzyloxy]benzoate (TAPER) to obtain a side-chain liquid crystalline copolymer (Figure 6a) [22]. The obtained random copolymer exhibited a columnar hexagonal mesophase with a clearing temperature of 128 °C. The main goal of this work was to exploit the LC polymer’s ability to self-assemble into a helical structure due to π-π stacking between neighboring aromatic cores, thereby forming columnar structures with an inner channel. Mimicking nature, particularly the tobacco mosaic virus, this bioinspired construction facilitates the construction of channels with selectivity towards protons over other cations. The inner part of the columns, containing basic oxygen atoms, is expected to transport protons across the membrane due to the electron-donating nature of oxygen, allowing protons to be transported without the need for water. The proton transport measurements demonstrated remarkable proton permeability comparable to Nafion^®^N117, with a value of approximately 2 × 10^−6^ cm^2^/s. Using a similar approach, Zare and co-workers reported the synthesis of a side-chain liquid crystal copolymer based on poly(epichlorohydrin-co-ethylene oxide) (PECH-co-EO) as the polymer backbone and the dendron TAPER as the side chains, achieving modification degrees of PECH-co-EO equal to 20 and 40% (Figure 6b) [24,28]. The cation transport capability of these membranes was evaluated by means of proton permeability and linear sweep voltammetry (LSV) for different monovalent cations, including H^+^, Na^+^, K^+^, and Li^+^.

**Figure 7:**
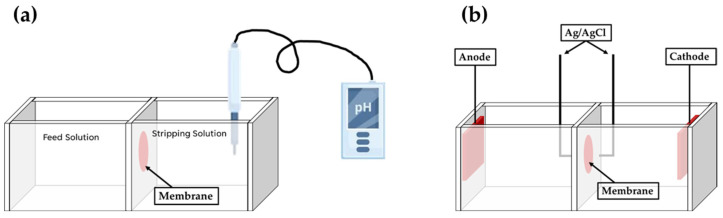
Experimental set-up of (**a**) permeability tests and (**b**) linear sweep voltammetry.

Proton permeability tests evaluate the transport of cationic species in both directions simultaneously using a two-compartment cell with the membrane placed between them (Figure 7a). In this test, a hydrochloric acid solution was added to the feed solution compartment, while a monovalent alkali metal ion solution (e.g., sodium chloride) was added to the stripping solution. The pH of the stripping solution is measured to monitor the proton transport from the feed solution into the stripping solution (Figure 7a). In this case, the proton permeability across the membrane can be calculated using Fick’s First Law [25].

LSV evaluates the transport of a single cationic species in one direction, driven by a potential difference (Figure 7b). In this experiment, the same solution was added to both compartments (the feed and stripping compartments), and we subsequently applied a potential difference to induce transport from one compartment to the other. The applied potential difference between the anode and cathode allows us to measure the electric current generated by this transport using Ag/AgCl electrodes (Figure 7b). As a result, we obtained a current–voltage curve that typically exhibits the following three regions: ohmic, limiting current, and overlimiting regions [29]. In the ohmic region, the current increases linearly with the potential, following an ohmic (or quasi-ohmic) relationship. This region is followed by the limiting current region, characterized by a plateau (or semi-plateau). The limiting current region is related to the permselectivity of the membrane and can or cannot be observed. Finally, after the limiting current region, the overlimiting region is observed, where the current typically increases with the potential. However, this final region is not related to any transport phenomenon but can be explained by the electroconvection theory.

Dendronized PECH-co-EO membranes evidenced the transport of H^+^, Na^+^, and Li^+^ in the permeability tests, while no transport of K^+^ was noticed. The authors suggested that the potassium cation was too large to permeate. The LSV measurements exhibited remarkable proton transport with a slightly higher selectivity for the protons compared to the other studied monovalent cations [28]. In order to elucidate how the polymer interacts with the cations during ion transport in this system (dendronized PECH-co-EO), Bogdanowicz and co-workers used in situ Raman spectroscopy to evaluate the transport phenomena. To carry out these experiments, chronoamperometry and Raman spectroscopy were combined to study a LC polyether with a low degree of modification with the dendron TAPER (a modification degree equal to 36%) as a model compound. Proton and sodium cations were used as model cationic species in the transport experiments. In the case of proton transport, the polyether backbone was found to be mainly involved in cation conductivity, as indicated by a change in the intensity of the band at 2870 cm^−1^. Additionally, an extra coordination site was observed, originating from the lateral oxygen of the carbonyl linker group, as evidenced by a change in the intensity of the band centered at 1250 cm^−1^. Furthermore, the presence of water within the system was proved not to influence cation transport across the membrane [118].

On the other hand, Ŝakalytė and co-workers prepared a family of polyamines based on poly[2-(aziridin-1-yl)ethanol] (PAZE) (Figure 6c), modified with the same dendron, aiming to improve proton conductivity due to the higher basic character of nitrogen atoms in the main chain [21,30]. This family of polyamines, whose members present different degrees of modification, ranging from 16 to 100%, all exhibited columnar mesophases. Bogdanowicz and co-workers used these polyamines to prepare ion-conducting membranes [23,29]. Nevertheless, assembling self-standing membranes proved to be nearly impossible due to their brittleness, which resulted in poor mechanical properties. In order to solve this drawback, an anodized aluminum oxide (AAO) support was used to improve the mechanical resistance of the membrane.

As aforementioned, a key advantage of these materials is their ability to build up columnar structures. However, this organization is not always well-oriented. In other words, these columnar structures are often randomly oriented. One of the goals of this work was to achieve homeotropic orientation of the polymer columns to create 1D parallel channels perpendicular to the membrane surface, thereby establishing effective conductive pathways. The use of a naturally oriented support like AAO, which has a honeycomb-like structure, should facilitate the orientation of the polymer columns and improve the mechanical resistance, as noted earlier. The hybrid membranes prepared with this support exhibited columnar mesophases and, in some cases, hexagonal columnar mesophases, as evidenced by X-ray diffraction [23]. Transport properties that were evaluated with LSV revealed remarkable proton transport compared to Nafion^®^117, along with outstanding proton selectivity over other monovalent cations (Na^+^, K^+^, and Li^+^). Permeability tests evidenced a similar behavior to that observed for the dendronized PECH-co-EO membranes: an antiport transport mechanism was confirmed for H^+^, Na^+^, and Li^+^, while no conductivity was denoted for K^+^ [29].

PAZE-based copolymers exhibit a strong tendency to crystallize, mostly due to their tendency to form hydrogen bonds. This tendency is consistent regardless of the degree of modification, from low degrees of modification up to 100% modified polymers. As a result, these polymers are difficult to orient and tend to be brittle. To overcome these drawbacks, Montané and co-workers explored the preparation of PAZE-modified copolymers containing both benzoyl groups and TAPER since this was expected to inhibit the crystallization tendency of these LCPs [26]. They found that the presence of benzoyl groups increased the clearing temperature of the system; however, hexagonal columnar mesophases were still observed. Homeotropically oriented membranes were successfully prepared, as evidenced by X-ray diffraction. Although the LSV data revealed that these membranes had lower selectivity compared to the previously reported PAZE-based membranes, they were easier to prepare, more cost-effective, had high thermal stability, and were not brittle [27].

As we have seen, all these TAPER-containing SCLCPs were obtained through a post-polymerization procedure involving the modification of commercial polyethers (PECH and PECH-co-EO) or synthesized polyamines (PAZE) with the dendronized mesogen TAPER. As a result, SCLCPs with different modification degrees were obtained, achieving degrees of modification up to 100% for PAZE and 72% for PECH-modified polymers. Using this methodology, copolymers were always obtained, except in the case of 100% modified PAZE, which resulted in a homopolymer. This homopolymer, however, exhibited a strong tendency to crystallize (probably due to its high symmetry) and had a low ability to form films.

With the aim to obtain dendronized side-chain LC homopolymers, Ŝakalytė, and co-workers synthetized an aziridine dendronized monomer [30]. However, polymerization of this tapered monomer was unsuccessful because the cleavage of the benzyl ether linkage took place, preventing effective polymerization.

**Figure 8:**
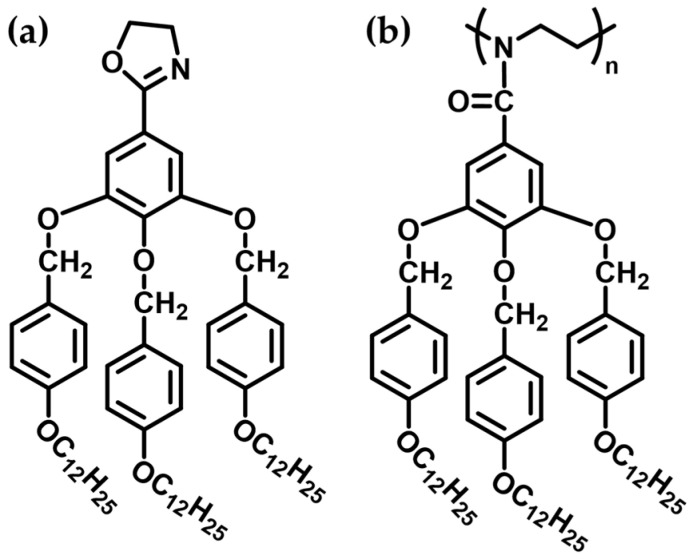
Chemical structure of (**a**) TAPOx and (**b**) PTOx.

To achieve this goal, Guardià and co-workers recently reported the synthesis of a 2-oxazoline dendronized monomer, 2-(3,4,5-tris(4-dodecyloxybenzyloxy) phenyl)-2-oxazoline (TAPOx, Figure 8a) [31], which was polymerized via living cationic ring-opening polymerization (CROP) [32]. The authors reported the synthesis of a family of poly(2-oxazoline) (PTOx, Figure 8b) with different degrees of polymerization ranging from 20 to 60, achieving good yields and low dispersity values (1.16–1.35), as determined by size exclusion chromatography (SEC). The reported homopolymers exhibited columnar mesophases, as evidenced by X-ray diffraction. PTOx, with a degree of polymerization of 40, formed a hexagonal columnar mesophase. Unlike PAZE-modified LCPs, this new LC homopolymer family did not tend to crystallize, with clearing temperatures of around 80 °C observed, according to DSC. Therefore, this new class of dendronized liquid crystal homopolymers are promising candidates for building up LC membranes for proton transport applications.

In summary, our research group reported the chemical modification of polyethers and polyamines with TAPER to obtain families of side-chain liquid crystalline polymers whose members present different modification degrees of TAPER. The resulting copolymers were used to prepare biomimetic membranes, some of which exhibited proton transport comparable to Nafion^®^, which is the benchmark material for their proton-conducting properties. The presence of oxygen or nitrogen atoms in the polymer backbone facilitates the transport of ions across the polymer’s helical structure. Nevertheless, further research is needed to improve the mechanical properties of these materials.

## 5. Conclusions

To sum up, the unique properties of liquid crystals and liquid crystal polymers, such as their ability to form organized mesophases, make them excellent candidates for different advanced materials, including optoelectronics devices, security inks, photovoltaics, biomedical applications, sensors, biosensors, etc.

In the field of membrane technology, research over the past few decades has shown that LCPs can be used to prepare membranes for ion-transport applications, wastewater treatment, and separation processes. For instance, in separation applications, these LC-based membranes have improved some features, such as pore homogeneity. Furthermore, when tested in fuel cells, LCP-based membranes have demonstrated enhanced selectivity compared to Nafion^®^.

Future research directions include designing new LC-based membranes with higher mechanical performance and enhanced resistance to fouling or biofouling. Moreover, improving organic photovoltaic devices is crucial for advancing toward a more sustainable world where LCs could play a key role. Biomedical applications could also lead to the development of new materials for controlled drug release, such as for cancer treatment, as well as for the formation of prosthetics and artificial muscles.

In conclusion, liquid crystals represent a dynamic and evolving field with a wide range of impactful applications. Moreover, continued interdisciplinary collaboration and innovation are essential to unlock the full potential of liquid crystals, paving the way for next-generation technologies that leverage their unique properties.
